# Affective dynamics and emotional reactivity in social anxiety disorder

**DOI:** 10.1017/S0033291725000121

**Published:** 2025-08-26

**Authors:** Beth Foote, Femke Lamers, Mike Xiao, Lihong Cui, Vadim Zipunnikov, Mathilde M. Husky, Kathleen R. Merikangas

**Affiliations:** 1 Department of Psychology, George Mason University, Fairfax, VA, USA; 2 Department of Psychiatry, Amsterdam UMC, Vrije Universiteit, Amsterdam Public Health Research Institute, Amsterdam, The Netherlands; 3 Child Mind Institute, New York, USA; 4 Genetic Epidemiology Research Branch, Intramural Research Program, National Institute of Mental Health, Bethesda, MD, USA; 5 Department of Biostatistics, Johns Hopkins Bloomberg School of Public Health, Baltimore, MD, USA; 6 Bordeaux Population Health Research Center, University of Bordeaux, Bordeaux, France

**Keywords:** social anxiety disorder, Ecological Momentary Assessment, reactivity, affective dynamics, events

## Abstract

**Background:**

Although heightened anxiety associated with social interaction or evaluation is the core diagnostic criterion for social anxiety disorder (SAD), there is growing evidence that SAD is characterized by more pervasive reactivity beyond social situations. We employed Ecological Momentary Assessment (EMA) to describe the affective dynamics and emotional reactivity to daily events in a community-based sample of adults with SAD compared with other anxiety disorders, and controls without anxiety or mood disorders.

**Methods:**

A sample of 236 adults with a lifetime diagnosis of SAD (n = 53), other anxiety disorders (n = 120), and no mood or anxiety disorder (n = 63) based on comprehensive diagnostic interviews answered brief electronic interviews that assessed daily life events and mood and anxiety symptoms four times a day for two weeks. Linear mixed models were used to quantify reactivity to daily life events.

**Results:**

Persons with SAD had higher average levels of sad and anxious mood than those with other anxiety disorders or controls. Irrespective of comorbid mood disorders, people with SAD also demonstrated significantly greater decreases in both sad and anxious mood following positive events, and a greater increase in anxious mood following negative, particularly non-social events.

**Conclusions:**

Our findings regarding pervasive reactivity beyond the social context in people with SAD confirm the need for broader conceptualization of this disorder as well as expansion of interventions beyond the social context. This work also demonstrates the utility of EMA as a powerful tool to track individual variability and reactivity in daily life that can inform etiology, treatment and prevention.

## Introduction

Social anxiety disorder (SAD) affects up to 12% of the population and is associated with significant personal and societal burdens (Ballard et al., 2019; Dalrymple & Zimmerman, 2007; Kessler et al., 2005). Characterized by increased sensitivity to social contexts involving the scrutiny or judgment of others, people with this disorder tend to avoid such situations or endure them with intense distress (American Psychiatric Association, 2013; Heimberg et al., 2014). However, it has been difficult to identify specific treatment targets for SAD and its consequences because of the pervasive comorbidity of SAD with other anxiety disorder subtypes and mood disorders that have been documented in both clinical and community samples (Stein et al., 2017).

Aside from the disabling impact of social anxiety on life functioning, suicide attempts, and substance use disorders are also well-established consequences of SAD in adults (Compton, Thomas, Stinson, & Grant, 2007; Cougle, Keough, Riccardi, & Sachs-Ericsson, 2009; Swendsen et al., 2010; Thibodeau, Welch, Sareen, & Asmundson, 2013) and youth (Leigh, Chiu, & Ballard, 2023; Lemyre, Gauthier-Legare, & Belanger, 2019). Our earlier work on the familial aggregation and co-aggregation of suicide attempts with mood and anxiety disorders showed that SAD was associated with an elevated risk of suicide attempts in both individuals and families of people with SAD, beyond the effects of comorbid mood and substance use disorders (Ballard et al., 2019). We speculated that while bipolar disorder, particularly comorbid with substance use disorders, was the most potent correlate of suicide attempts, SAD may reflect a proximate trigger of suicide attempts through increased reactivity to acute life stressors. Here, we examine this potential mechanism by examining patterns of daily reactivity and variability of mood and anxiety with ecological momentary assessment (EMA).

Previous studies have demonstrated that people with SAD may have broader reactivity to life events beyond those involving social appraisal. Studies using self-reported or physiologic measures have shown that people with SAD have increased reactivity to social events in general (Barker, Troller-Renfree, Pine, & Fox, 2015; Goldin, Manber, Hakimi, Canli, & Gross, 2009; Kagan, Reznick, & Snidman, 1987; Nelemans et al., 2017; Roelofs, Minelli, Mars, van Peer, & Toni, 2009; Yoon & Joormann, 2012) as well as to social stressors, neutral events (Bowen, Baetz, Hawkes, & Bowen, 2006; Farmer & Kashdan, 2015; Kashdan & Collins, 2010) and even positive events such as praise (Clark & Wells, 1995; Doorley et al., 2021; Hur, Tillman, Fox, & Shackman, 2019; Rapee & Heimberg, 1997). Some studies suggest that SAD may reflect broader mood alterations or vulnerabilities that extend beyond the social context (Auerbach, Richardt, Kertz, & Eberhart, 2012; Kashdan & Collins, 2010; Piccirillo & Rodebaugh, 2022). However, few of the earlier studies of reactivity of SAD included comprehensive diagnostic interviews of the full range of psychopathology that could characterize comorbid conditions such as mood disorders which are also associated with both event reactivity as well as general affective dynamics (Lamers et al., 2018). Moreover, most of the evidence of reactivity in SAD has been based on either unselected or clinical samples of young adults.

The present study examines the real-time affective dynamics and emotional reactivity to daily life events in a community-ascertained sample of a broad age range of adults who were characterized by the full range of anxiety and mood disorder subtypes with comprehensive diagnostic assessments. The specific aims are to: (1) evaluate the differences, variability, and instability of sad and anxious moods in people with SAD, other anxiety and/or mood disorders, and controls without mood or anxiety disorders; (2) examine whether there is enhanced mood reactivity to both negative and positive events among people with and without SAD, and (3) determine whether there is an interaction between social versus non-social event type with mood reactivity.

## Methods

### Sample

A sample of 236 individuals 18 years and older participated in this study as part of the National Institute of Mental Health (NIMH) Family Study of Affective Spectrum Disorders, a large family study based on DSM-IV-TR mood and anxiety disorders. Probands were recruited from the greater Washington DC metropolitan area and enriched for mood disorders through referrals from the National Institute of Health Clinical Center or the National Institute of Mental Health Mood and Anxiety Disorders Program. The community sample, designed to be a nontreatment or nonclinical group with and without mental health disorders, was ascertained by mail contact via a list of households within a 50-mile radius of Washington, DC. Participants who were recruited from the community could be currently in treatment or have been previously treated for their mental health problems or disorders. Inclusion criteria were the ability to speak English, availability to participate, and consent to contact at least two living relatives. All participants provided written informed consent, and the study was approved by the Combined Neuroscience IRB at the National Institutes of Health. Additional details of the family study methods are described elsewhere (Merikangas et al., 2014).

We compared EMA-based emotional ratings between those with a lifetime SAD (n = 53), other lifetime anxiety disorders (n = 120), and no lifetime mood or anxiety disorders (n = 63). Of the participants with lifetime SAD, 42 (79%) met criteria for current SAD. Of the 53 individuals with lifetime SAD, 37 (69.8%) also had a lifetime history of generalized anxiety disorder (GAD) and/or panic disorder, 23 (43.4%) with specific phobia, and 46 (86.8%) with a mood disorder (Bipolar I, Bipolar II, or Major Depressive Disorder). Dysthymia was not incuded as a separate disorder because of overlap with one of the mood disorder subtypes. Individuals in the ‘other anxiety disorder’ group most frequently had GAD or panic disorder (n = 73; 60.8%), specific phobia (n = 59; 49.2%), or other anxiety disorders (separation anxiety or agoraphobia) (n = 14; 49.2%). Panic disorder and GAD were combined as only 22 subjects in the entire sample presented with lifetime panic disorder and 73% of those with panic disorder also met the criteria for lifetime GAD.

### Measures

#### Diagnostic interview

The NIMH Family Study Diagnostic Interview for Affective Spectrum Disorders ascertains diagnostic criteria as well as subthreshold phenomenology related to current and lifetime disorders from the Diagnostic and Statistical Manual of Mental Disorders (DSM-IV). The interview was developed based on earlier diagnostic interviews for genetic epidemiologic studies such as the Schedule for Affective Disorders and Schizophrenia and the Diagnostic Interview for Genetic Studies, and does not adhere to skip-outs based on frequency or duration at the probe level. Experienced clinicians conducted direct interviews of probands and direct, blinded interviews of relatives. Inter-rater reliability of all diagnostic categories was high, with intraclass correlations of 0.87 and above across all diagnostic categories. Best estimate DSM-IV diagnoses were based on all available information, including direct interviews, family history, and consensus ratings from experienced clinicians. Family members of the proband were interviewed about their own mental health as well as that of their relatives. More detailed information concerning the semi-structured interview and family study methods can be found in an earlier publication of this work (Merikangas et al., 2014).

#### EMA procedures

Participants completed brief electronic questionnaires four times a day over a 2-week period using a Personal Digital Assistant (Tungsten E2 PDA). Fixed assessment times were used for each participant with an average delay of 4 hours between assessments, and signaling schedules varied across participants. Participants completed EMA within the 12 months following the clinical interview (and the majority within the first 2 months following the clinical assessments) and were trained and monitored by research assistants on administrative procedures. The NIMH EMA was subsequently administered on research mobile devices and more recently is available through an open-source platform (Klein et al., 2021).

The EMA variables examined in the present study included mood states based on the mood circumplex model (Larsen & Diener, 1992) and daily events based on the inventory of small life events that was adapted for use in EMA (Lamers et al., 2018; Zautra, Guarnaccia, & Dohrenwend, 1986). The circumplex model of affect describes emotional experiences using orthogonal dimensions reflecting valence (pleasant to unpleasant) and activation (high to low activation) and their combination (Feldman-Barrett & Russell, 1998; Larsen & Diener, 1992; Posner, 2005; Russell, 1980; Yik, 1999). Larsen and Diener’s (Larsen & Diener, 1992) mood circumplex model further proposes four bipolar scales reflecting the full circumplex structure of affective states. Participants were asked to rate their anxious mood at the time of the EMA assessment on a Likert scale from (1) very calm to (7) very anxious, and their sad mood from (1) very happy to (7) very sad. Other mood states were also assessed through EMA but were not examined in the present study. For daily events, participants were first asked to identify the event that affected them the most since the previous assessment, and then to rate the impact of the event on a Likert scale from (1) extremely positive to (7) extremely negative. This impact variable was then categorized into positive events (a score of 1–3), neutral events (score of 4), and negative events (scores of 5–7). In addition, participants were asked to classify the type of event they had experienced with the option of choosing any of the following: education, family or friends relationships, interactions with colleagues, interactions with strangers, housing or residence, leisure, health, finances, religion or spirituality, legal or judicial, traveling or commuting, after school activities, interactions with students, or other events. Participants were instructed to identify one category (out of a list of 14 mutually exclusive types of events) that reflected the nature of the event they identified at each assessment. They were provided examples during training to elicit unpleasant events at work as either ‘interaction with colleagues’ or as a ‘work’ event if it was not specifically related to interpersonal relationships. These event types were then categorized according to their social (family and friends relationships, or interactions with colleagues, students, or strangers) or non-social nature (all other events).

#### Statistical analyses

Affective dynamic analyses were conducted in SAS version 9.4 (SAS Institute Inc., Cary, NC) using the Generalized Least Squares (GLS) method and Restricted Maximum Likelihood (REML) to estimate the fixed effects and the covariance parameters, respectively. The outcome variables used in the models were mean analog mood rating level, mood variability, and instability for the spectrums of both anxious and sad mood states (resulting in six models). These effects were modeled with the fixed effect of anxiety disorder lifetime status and the reference group included non-SAD anxiety disorders (Other Anxiety), and Controls without lifetime mood and anxiety disorders. The models adjusted for the presence of lifetime-specific phobia, GAD, or panic disorder to investigate the specificity of the findings regarding SAD versus other anxiety disorder subtypes. Mood variability was based on the within-day average standard deviation (SD) of assessments, and instability was defined as the average within-day mean squared successive difference (MSSD) and was transformed using ln (MSSD + 1) to a normal distribution. The following covariates were used in the affective dynamic models: age, sex, lifetime anxiety or mood disorder, and Global Assessment of Functioning (GAF) score.

For all analyses on event reactivity SPSS version 22 and linear mixed models, with random intercepts and a repeated effect with an AR (1) correlation structure was used to account for multiple observations per person. This method allows us to fully harness the power of repeated measures in our data. Compliance was high for the sample with participants completing an average of 44 (SD 9.9; 77.8%) of the 56 programmed assessments. All data were used with no restrictions on a minimum number of EMA assessments completed due to high compliance missing assessments were not imputed. Reactivity to positive and negative events was examined through two models with the outcome variable of mood state (sad or anxious) and fixed effects of lifetime diagnosis status, event valence (positive or negative events, with neutral events as the reference group), interaction of SAD and event type, and with adjustment for age, sex, comorbid mood disorder, GAF score, and mood rating at the previous assessment. The analyses for reactivity to positive and negative events were conducted separately for social events and non-social events and had the following fixed effects of anxiety diagnoses, event valence (positive, negative, neutral as the reference group), mood rating at the previous assessment, and the interactions of both SAD and event valence. For more information concerning multilevel modeling (see Twisk, 2006).

## Results

Table 1 presents the sociodemographic clinical characteristics of the three study groups included in the sample The sample included 236 participants, 62.7% of whom were female with an average age of 48.2 years (SD = 16.2, range = 18–84). The sex distribution of individuals with SAD did not differ from those with other anxiety disorders and controls. However, controls were older than the case groups and there were fewer female controls (41.3%) than cases of SAD (64.2%) or other anxiety disorders (74.2%). Participants with SAD were more likely to have a current diagnosis compared with those with other anxiety disorders and they had significantly lower GAF scores than the combined reference group (t = −7.935, p < .001), the other anxiety disorder (t = −5.144, p < .001) and the control groups (t = −13.363, p < .001). The other anxiety group had significantly lower GAF scores than the control group (t = 11.07, p < .001).

**Table 1.** Demographic and clinical characteristics of sample by lifetime anxiety and mood disorders

Diagnostic group						
Variables	Social anxiety	Other anxiety disorder	Controls	Total	Test statistic	P value
N	53	120	63	236		
Female sex, (%)	34 (64.2)	89(74.2)	26(41.3)	148 (62.7%)	X2 (2) =1077.4	**<.0001**
Mean age, (SD)	46.7 (13.9)	47.0(15.3)	51.7(18.9)	48.2 (16.2)	KW (2) =167.8	**<.0001**
Current disorder N, (%)	50(96.2)	98 (82.4)	NA	148(62.7)	X2 (1) =5.9207	**0.015**
Mean GAF score (SD)	61(10)	68.15(7.6)	79.7(4.5)	69.6 (10.1)	KW(2) = 118.71	**<.0001**
**Lifetime disorders**						
Specific phobia N, (%)	23 (43.4)	59 (49.2)	NA	82 (34.7)	X2 (1) =27.264	**<.0001**
GAD/panic disorder N, (%)	37 (69.8)	73 (60.8)	NA	110 (46.6)	X2 (1) =71.279	**<.0001**
Mood disorder N, (%)	46 (86.8)	89 (74.2)	NA	135 (57.2)	X2 (1) =190.73	**<.0001**

*Note:* The reference group includes participants who have an anxiety disorder but do not have SAD and controls who do not meet the criteria for a mental disorder. The current disorder is defined as any mood and anxiety disorder in the prior year of the date of the interview. SAD: Social Anxiety Disorder. GAD: Generalized Anxiety Disorder. Mood disorder is a lifetime diagnosis of Bipolar I, Bipolar II, or Major Depressive Disorder. GAF: Global Assessment of Functioning. Kruskal–Wallis Test (KW).

Participants completed an average of 44 (SD = 9.9; 77.8%) of the 56 programmed assessments. People with SAD were less compliant than controls in completing an average of 41 assessments (SD = 12.5; 74%), t = 2.042, p < .05. There was an average of 8.3 neutral events (SD = 7.4), 27.6 positive events (SD = 12.2), and 7.7 negative events (SD = 6.9) across the assessment period. Of all reported events, there were approximately equal proportions of events that were described as having a social (M = 20, SD = 10.6) or non-social nature (M = 23.3, SD = 10.6). There were no significant differences in the frequency of neutral events, or type of event, between individuals with SAD compared with controls. However, people with SAD reported significantly fewer positive events (t = 4.931, p < .001) and significantly more negative events (t = 3.977, p < .001) than the combined reference group. In addition, people with SAD reported significantly fewer positive events than the other anxiety disorder group and controls, (F = 13.239, p < .001), while there was no difference between controls and the other anxiety disorder group. Likewise, people with SAD reported significantly more negative events than both the other anxiety group and controls, and the other anxiety group had significantly more negative events than controls (h [Kruskal–Wallis] = 26.949, p < .001).

### Intensity, variability, and instability of sad and anxious moods

There was a moderate level of variability of both sad mood (SAD group M = 1.1, SD = 0.3; other anxiety disorder group M = 2.8, SD = 0.9; and control group M = 2.4, SD = 0.8), and anxious mood (SAD group M = 3.2, SD = 1; other anxiety disorder group M = 2.5, SD =0.9; and control group M = 2, SD = 0.7) across the 2-week period. The affective dynamics linear mixed models by lifetime disorder status are presented in Table 2. After adjustment for mood disorders, only individuals with lifetime SAD had significantly greater sad mood (coef = 0.526, p < .001). Likewise, the presence of lifetime SAD was significantly associated with increased anxious mood (coef = 0.381, p < .05), as was the presence of lifetime GAD/Panic disorder (coef = 0.369, p = 0.009). The findings of greater sad (coef = 0.482, p < .01) and anxious (coef = 0.350, p < .05) mood remained significant when including only individuals with a current SAD diagnosis. Furthermore, there were no differences in the variability or instability of sad and anxious mood among those with current SAD. However, those with GAD/Panic disorder also had significantly greater instability in anxious mood (coef = 0.128, p = 0.04).

**Table 2.** Within-day means, variability, and instability of sad and anxious mood in individuals by lifetime anxiety disorder status

					SAD MOOD d,t				
	Average mood (Within-day Mean)			Mood variability (Average within-day SD)			Mood instability (Average within-day MSSD) †		
Variables	Estimate	Confidence interval	P	Estimate	Confidence interval	P	Estimate	Confidence interval	P
**Lifetime disorders**									
SAD	**0.526**	**(0.47, 1.03)**	**<.001**	0.047	(−0.04, 0.14)	0.291	0.039	(−0.06, 0.14)	0.433
Specific phobia	−0.126	(−0.36, 0.11)	0.288	0.029	(−0.05, 0.11)	0.444	0.013	(−0.07, 0.10)	0.763
GAD/Panic disorder	−0.015	(−0.30, 0.27)	0.918	0.055	(−0.04, 0.14)	0.229	0.048	(−0.05, 0.15)	0.349
**Covariates**									
Mood disorder	−0.0002	(−0.32, 0.31)	0.999	0.065	(−0.04, 0.17)	0.203	0.061	(−0.05, 0.17)	0.285
Sex (F v.s M)	−0.164	(−0.39, 0.06)	0.150	**0.092**	**(0.02, 0.17)**	**0.013**	**0.094**	**(0.01, 0.18)**	**0.023**
Age	−0.005	(−0.01, 0.00)	0.104	−0.001	(−0.003, 0.00)	0.381	−0.0003	(−0.003, 0.002)	0.810
GAF Score	−0.034	(−0.05,–0.02)	<.0001	0.002	(−0.004, 0.01)	0.570	0.001	(−0.005,0.01)	0.802

					ANXIOUS MOOD d,t				
	Average mood (Within-day Mean)			Mood variability (Average Within-day SD)				Mood instability (Average Within-day MSSD†)	
Variables	Estimate	Confidence interval	P	Estimate	Confidence interval	P	Estimate	Confidence interval	P
**Lifetime disorders**									
SAD	**0.381**	**(0.09, 0.66)**	**0.01**	0.049	(−0.07, 0.17)	0.424	0.053	(−0.08, 0.18)	0.417
Specific phobia	0.050	(−0.19, 0.29)	0.677	0.041	(−0.06, 0.14)	0.411	0.038	(−0.07, 0.14)	0.462
GAD/Panic disorder	**0.369**	**(0.09, 0.65)**	**0.009**	**0.163**	**(0.05, 0.28)**	**0.005**	0.122	(−0.003, 0.25)	0.050
**Covariates**									
Mood disorder	−0.006	(−0.32, 0.31)	0.970	0.089	(−0.04, 0.22)	0.173	0.119	(−0.02, 0.26)	0.089
Sex	−0.190	(−0.42, 0.04)	0.100	0.032	(−0.06, 0.13)	0.501	0.031	(−0.07, 0.13)	0.542
Age	**−0.007**	**(−0.14, −0.00)**	**0.030**	−0.002	(−0.005, 0.00)	0.130	−0.002	(−0.005, 0.0006)	0.107
GAF score	**−0.026**	**(−0.04,–0.01)**	**0.002**	0.002	(−0.005, 0.01)	0.627	0.002	(−0.01, 0.01)	0.590

*Note:* All disorders are lifetime diagnoses. Mood disorder includes either Bipolar I, Bipolar II, or Major Depressive Disorder. SAD: Social Anxiety Disorder; GAF: Gobal Assessment of Functioning; †MSSD = mean square successive difference had the following transformation: ln(mssd+1), SD = standard deviation. Linear mixed model adjusted for age, sex, and comorbid mood disorders. Outcome-based on sad and anxious mood rating at each assessment (d, t). Confidence intervals are 95%. Significant findings are marked in bold.

### Mood reactivity to positive and negative events

Table 3 shows the associations between mood reactivity and the valence of life events by the presence or absence of lifetime disorders. There were significant main effects for the associations between both positive and negative events, with decreases in sad and anxious moods following a positive event and increases following a negative event. There were statistically significant interactions for positive events for sad mood and both positive and negative events for anxious mood among those with SAD. These interactive influences reflected a greater decrease in both sad mood (slope = −0.687) and anxious mood after positive events (slope = −0.608) for those with SAD compared with controls, and a greater increase in anxious mood following negative events (slope = 0.769).

**Table 3.** Reactivity of ratings of sad and anxious mood following daily events by lifetime disorder status

		Sad moodd,t			Anxious moodd,t	
Variables	Estimate	Confidence interval	P value	Estimate	Confidence interval	P value
**Significant events**						
Neutral event	ref			ref		
Positive event	**−0.530**	**(−0.60, −0.46)**	**<.0001**	**−0.410**	**(−0.49, −0.33)**	**<.0001**
Negative event	**0.555**	**(0.46, 0.65)**	**<.0001**	**0.529**	**(0.42, 0.64)**	**<.0001**
**Lifetime disorders**						
SAD	**0.250**	**(0.052, 0.45)**	**0.013**	0.196	(−0.03, 0.42)	0.083
Specific phobia	−0.115	(−0.25, 0.02)	0.093	0.049	(−0.10, 0.20)	0.514
GAD/panic disorder	−0.044	(−0.21, 0.12)	0.595	**0.276**	**(0.10, 0.45)**	**0.003**
**Covariates**						
Mood disorder	−0.041	(−0.21, 0.13)	0.645	−0.007	(−0.20, 0.19)	0.946
Sex (F vs. M)	0.106	(−0.02, 0.24)	0.110	**0.152**	**(0.01, 0.30)**	**0.040**
Age	−0.001	(−0.01, 0.01)	0.466	−0.003	(−0.01, 0.00)	0.117
GAF score	**−0.027**	**(−0.04, −0.02)**	**<.0001**	**−0.016**	**(−0.02, −0.01)**	**0.001**
**Interactions: Event type by SAD**						
Positive by SAD	**−0.157**	**(−0.30, −0.01)**	**0.037**	**−0.198**	**(−0.37, −0.03)**	**0.023**
Negative by SAD	0.053	(−0.12, 0.22)	0.544	**0.240**	**(0.04, 0.44)**	**0.018**
Mood stated. t−1	**0.301**	**(0.28, 0.32)**	**<.0001**	**0.267**	**(0.255, 0.29)**	**<.0001**

*Note:* All disorders are lifetime diagnoses. Mood disorder is a lifetime diagnosis of Bipolar I, Bipolar II, or Major Depressive Disorder.SAD: Social Anxiety Disorder. GAF: Global Assessment of Functioning. Linear mixed model corrected for age, sex, GAF score, and mood at previous assessment (d, t−1). Significant findings are indicated in bold. Interaction with events only for SAD, other comorbid disorders are only corrected for as main effects. Outcome-based on sad and anxious mood rating at each assessment (d, t) and confidence interval are 95%.

### Mood reactivity to social and non-social events

We also evaluated the association between SAD with mood reactivity by the social significance of the event. Significant interactions were observed between event type and SAD. Individuals with lifetime SAD experienced greater decreases in sad (coef = −0.322, p < .001) and anxious mood (coef = −0.315, p < .01) following positive events, and a greater increase in anxious mood (coef = 0.353, p < .05) following negative events. However, these effects were observable only for non-social events. These differences remained significant when including only those individuals with current SAD.

## Discussion

Using real-time mobile tracking of emotions and events in a comprehensively characterized community-based sample, we found that SAD may be characterized by a broader range of emotional dysregulation than is recognized by current nosology. Specifically, patterns of affective dynamics and emotional reactivity to both positive and negative events in individuals with this disorder differed from those with other anxiety disorder subtypes and controls without a history of mood or anxiety disorders and persisted after controlling for a lifetime mood disorder. The findings indicate that SAD may be characterized by enhanced sensitivity and reactivity to daily perturbations beyond those of a social nature. The persistence of SAD across the lifespan as demonstrated by similar patterns of reactivity among those with either current or lifetime SAD also highlights the enduring nature of the temperamental sensitivity of this condition that distinguishes it from the more episodic manifestations of other anxiety disorders such as panic disorder and major depression. More broadly, this work demonstrates the utility of the application of real-time mobile technology to track emotional states in daily life that may enhance our ability to characterize the dynamics of SAD and define targets for interventions, that could ultimately inform suicide prevention (Farmer & Kashdan, 2015; Hur et al., 2020; Rashid, Chakraborty, & Fraser, 2020).

The greater average levels of both sad and anxious mood among those with SAD compared with those with other anxiety disorders and controls highlights its distinction from other anxiety disorders and further emphasizes the broad range of emotional manifestations of SAD (Farmer & Kashdan, 2015; Piccirillo & Rodebaugh, 2022). The persistence of these differences after adjustment for comorbid mood disorders suggests that elevated emotional arousal may comprise a core phenomenological trait in SAD (Rapee & Heimberg, 1997).

Our finding of significantly greater decreases in both sad and anxious moods following positive events in those with SAD, and greater increase in anxious mood following negative events regardless of event type further supports the expansion of definitions of SAD to reflect its broader emotional reactivity beyond the traditional conceptualization of greater sensitivity to social interactions (Clark & Wells, 1995; Doorley et al., 2021; Dryman & Heimberg, 2018; Hur, Stockbridge, Fox, & Shackman, 2019). Moreover, this increased reactivity of people with SAD was not attributable to comorbid mood disorders as shown by earlier EMA studies (Farmer & Kashdan, 2015; Piccirillo & Rodebaugh, 2022). These findings argue for a broader conceptualization of SAD that distinguishes this subgroup both from other anxiety disorder subtypes as well as from mood disorders.

The lack of a significant increase in anxiety following social events among those with SAD was unexpected, considering that increased anxiety in social situations is a core feature of this disorder. Potential explanations for this negative finding could be that there was overlap in the context, valence, impact, and social nature of the events that may have obscured these distinctions, or methodologic in which our instructions for EMA may have overemphasized the negative versus positive impact over-classification of the nature of the event. Furthermore, we did not collect detailed information on the social context in terms of familial versus unfamiliar people present at the time of the ratings so could not test this potential effect.

The present results have implications for understanding the course of SAD, treatment, and prevention of its consequences. The broad mood reactivity observed in these individuals may contribute to the lifetime persistence of this condition that distinguishes it from both mood and other anxiety disorders that tend to fluctuate across the lifespan (Angst, Gamma, Baldwin, Ajdacic-Gross, & Rössler, 2009; Bruce et al., 2005). Sad and anxious moods, albeit elevated, tended to be more stable in people with SAD because they did not exhibit the greater variability and instability of emotions that characterize people with mood disorders (Koval, Pe, Meers, & Kuppens, 2013; Lamers et al., 2018; Schwartz, Schultz, Reider, & Saunders, 2016). Moreover, the salience of mood symptoms as well as enhanced reactivity to daily events may play an important role in the increased rates of suicide attempts in people with SAD, particularly when comorbid with bipolar disorder (Ballard et al., 2019). Mechanistic studies that investigate the role of underlying traits such as behavioral inhibition (Clauss & Blackford, 2012), anxiety sensitivity (Stanley et al., 2018), and physiologic reactivity (Venables et al., 2018) in the link between interpersonal sensitivity and suicidal ideation and attempts (Buckner, Lemke, Jeffries, & Shah, 2017) are needed. Such reactivity should be particularly considered in the context of mood and substance use disorders that could facilitate the translation of these findings to prevention and interventions. Therefore, these broader manifestations of SAD should be incorporated into program content and measures of outcome in both adults and youth (Butler, O’Day, Swee, Horenstein, & Heimberg, 2021; Leigh & Clark, 2018).

The strengths of the present study include its use of a large community-based sample, the wide age range and inclusion of both male and female participants, the application of comprehensive diagnostic assessments that include the full range of mood and anxiety disorder subtypes and substance use disorders, and the balance between those with current versus lifetime disorders. Most prior EMA studies of SAD were either based on laboratory studies or convenience samples without a comprehensive diagnostic assessment. Adjustment of statistical models for comorbid mood disorders provided a more conservative investigation of the specificity of alterations in affective dynamics and event sensitivity in SAD. In addition, the control for emotional states before event occurrence permitted a highly precise examination of the direction of associations among mood states and daily events.

However, the present findings should also be interpreted relative to specific limitations or characteristics of the methods. First, the analyses assessed within-day associations among emotions and events over a 2-week period. While these timeframes correspond to the natural trajectories of mood changes following daily events (Johnson et al., 2008), analyses over longer timeframes may produce different results. Second, as described above, the lack of specificity of reactivity to social events may in part be the misclassification of distinctions between social and non-social events in the EMA ratings. Third, participants were asked to rate the positive or negative ‘impact’ of each event, reflecting both the level of disruption caused by the event as well as its valence. This may have confounded valence and impact. However, similar methods have been used in prior EMA studies assessing individuals’ experience of daily life stressors (Husky et al., 2009; Johnson et al., 2008; Khazanov et al., 2019; Lamers et al., 2018). Fourth, our findings are focused on an adult sample, and therefore may not apply to SAD in children and adolescents. Although our sample is older than prior clinical samples, we observed similar results for reactivity to negative events as those of previous studies (Farmer & Kashdan, 2015). Moreover, although the findings are based on lifetime SAD, there is abundant evidence that the core feature of social sensitivity tends to persist across the lifetime irrespective of fluctuations in the salience of its associated features and impairment (Bruce et al., 2005; Kessler, Petukhova, Sampson, Zaslavsky, & Wittchen, 2012). One important topic for future research is whether SAD may reflect a lack of maturation of either physiologic or cognitive reactivity to environmental events that could be a target for developmentally sensitive interventions (Richey et al., 2019). Additionally, future studies should include greater specificity for characterizing events as social or non-social to capture more nuanced reactivity patterns to different social contexts.

## Conclusion

People with SAD from a non-clinical community-based sample had elevated levels of both sad and anxious mood, and enhanced reactivity to both positive and negative daily events, regardless of the social nature of the event. This increase in general reactivity that tends to persist across the lifetime suggests that expansion of interventions for SAD beyond the social context may be more effective in reducing its negative life consequences. Findings support the application of tools to track daily fluctuations among those with enhanced vulnerability to suicide as a preventive intervention. More broadly, this work further demonstrates the utility of EMA as a powerful tool to track individual variability and reactivity in daily life that can inform etiology, treatment, and prevention.

## Supporting information

Foote et al. supplementary material Foote et al. supplementary material

## Supplementary material

The supplementary material for this article can be found at http://doi.org/10.1017/S0033291725000121.
