# Supplementary material for: Affective dynamics and emotional reactivity in social anxiety disorder
Source: Psychol Med. 2025 Aug 26;55:e242. doi: 10.1017/S0033291725000121 (PMC12404326; doi:10.1017/S0033291725000121)
Supplement: Foote et al. supplementary material [file S0033291725000121sup001.docx]

# **Social Anxiety Disorder and Emotional Reactivity**

# **Supplemental Table S-1.** Emotional reactivity to significant events among those with Lifetime Anxiety Disorder Subtypes stratified by social context

| **Social Events**  **Sad Moodd,t Anxious Moodd,t** | | | | | |  | **Not Social Events**  **Sad Moodd,t Anxious Moodd,t** | | | | |  |
| --- | --- | --- | --- | --- | --- | --- | --- | --- | --- | --- | --- | --- |
| **Variables** | **Estimate** | **Confidence Interval** | **p** | **Estimate** | **Confidence Interval** | **p** | **Estimate** | **Confidence Interval** | **p** | **Estimate** | **Confidence Interval** | **p** |
| **Significant Events** | | | | | | | | | | | | |
| Neutral event | ref |  |  | ref |  |  | ref |  |  | ref |  |  |
| Positive event | **-0.691** | **(-0.81, -0.57)** | **<.0001** | **-0.529** | **(-0.67, -0.39)** | **<.0001** | **-0.438** | **(-0.53, -0.35)** | **<.0001** | **-0.325** | **(-0.43, -0.22)** | **<.0001** |
| Negative event | **0.706** | **(0.55, 0.86)** | **<.0001** | **0.483** | **(0.53, 0.89)** | **<.0001** | **0.414** | **(0.29, 0.53)** | **<.0001** | **0.387** | **(0.24, 0.53)** | **<.0001** |
| **Lifetime Disorder** | **s** |  |  |  |  |  |  |  |  |  |  |  |
| SAD | 0.112 | (-0.15, 0.37) | .394 | **0.235** | **(-0.07, 0.54)** | **.127** | **0.348** | **(0.12, 0.57)** | **.003** | 0.189 | (-0.06, 0.44) | .132 |
| Specific phobia | **-0.199** | **(-0.33, -0.07)** | **.004** | -0.031 | (-0.19, 0.13) | .701 | -0.025 | (-0.18, 0.13) | .744 | 0.114 | (-0.04, 0.27) | .158 |
| GAD/Panic disorder | -0.037 | (-0.20, 0.12) | .642 | **0.218** | **(0.03, 0.41)** | **.025** | -0.002 | (-0.19, 0.18) | .984 | **0.295** | **(0.10, 0.49)** | **.003** |
| **Covariates** |  |  |  |  |  |  |  |  |  |  |  |  |
| Mood disorder | -0.039 | (-0.21, 0.13) | .657 | 0.071 | (-0.13, 0.28) | .494 | -0.059 | (-0.26, 0.14) | .556 | -0.033 | (-0.24, 0.17) | .750 |
| Sex | 0.125 | (-0.01, 0.26) | .062 | 0.157 | (-0.001, 0.31) | .051 | 0.113 | (-0.03, 0.26) | .129 | **0.172** | **(0.02, 0.32)** | **.027** |
| Age | -0.001 | (-0.005, 0.00) | .628 | -0.003 | (-0.01, 0.00) | .167 | -0.002 | (-0.01, 0.00) | .465 | -0.004 | (-0.01, 0.00) | .104 |
| GAF score | **-0.026** | **(-0.03, -0.02)** | **<.0001** | **-0.014** | **(-0.02, -0.004)** | **.009** | **-0.027** | **(-0.04, -0.02)** | **<.0001** | **-0.015** | **(-0.03, -0.004)** | **.005** |
| **Interactions: Event Type by SAD** | | | | | | | | | | | | |
| Positive by SAD | 0.043 | (-0.20, 0.29) | .731 | -0.112 | (-0.39, 0.17) | .432 | **-0.322** | **(-0.51, -0.13)** | **.001** | **-0.315** | **(-0.54, -0.09)** | **.006** |
| Negative by SAD | -0.003 | (-0.28, 0.27) | .985 | 0.066 | (-0.25, 0.38) | .680 | 0.034 | (-0.20, 0.27) | .775 | **0.353** | **(0.08, 0.63)** | **.011** |
| Mood stated. t-1 | **0.315** | **(0.28, 0.35)** | **<.0001** | **0.228** | **(0.20, 0.26)** | **<.0001** | **0.294** | **(0.26, 0.32)** | **<.0001** | **0.309** | **(0.28, 0.34)** | **<.0001** |

All disorders are lifetime diagnoses. Mood disorder is a lifetime diagnosis of Bipolar I, Bipolar II, or Major Depressive Disorder. GAF (Global Assessment of Functioning). Models adjusted for age, sex, GAF score, and mood at previous assessment (d, t-1). Significant findings indicated in bold. Interactions only for SAD and event type. Outcome based on sad and anxious mood rating at each assessment (d, t) and confidence interval are 95%.
